# Inertial Sensor-Based Gait and Attractor Analysis as Clinical Measurement Tool: Functionality and Sensitivity in Healthy Subjects and Patients With Symptomatic Lumbar Spinal Stenosis

**DOI:** 10.3389/fphys.2018.01095

**Published:** 2018-08-14

**Authors:** S. Kimberly Byrnes, Corina Nüesch, Stefan Loske, Andrea Leuenberger, Stefan Schären, Cordula Netzer, Annegret Mündermann

**Affiliations:** ^1^Clinic for Orthopaedics and Traumatology, University Hospital Basel, Basel, Switzerland; ^2^Department of Biomedical Engineering, University Hospital Basel, Basel, Switzerland; ^3^Clinic for Spinal Surgery, University Hospital Basel, Basel, Switzerland; ^4^Department of Clinical Research, University Hospital Basel, Basel, Switzerland

**Keywords:** IMU, 6MWT, gait variability, gait changes, decompression surgery

## Abstract

**Objective:** To determine if the attractor for acceleration gait data is similar among healthy persons defining a reference attractor; if exercise-induced changes in the attractor in patients with symptomatic lumbar spinal stenosis (sLSS) are greater than in healthy persons; and if the exercise-induced changes in the attractor are affected by surgical treatment.

**Methods:** Twenty-four healthy subjects and 19 patients with sLSS completed a 6-min walk test (6MWT) on a 30-m walkway. Gait data were collected using inertial sensors (RehaGait^®;^) capturing 3-dimensional foot accelerations. Attractor analysis was used to quantify changes in low-pass filtered acceleration pattern (δM) and variability (δD) and their combination as attractor-based index (δF = δM^*^ δD) between the first and last 30 m of walking. These parameters were compared within healthy persons and patients with sLSS (preoperatively and 10 weeks and 12 months postoperatively) and between healthy persons and patients with sLSS. The variability in the attractor pattern among healthy persons was assessed as the standard deviation of the individual attractors.

**Results:** The attractor pattern differed greatly among healthy persons. The variability in the attractor between subjects was about three times higher than the variability around the attractor within subject. The change in gait pattern and variability during the 6MWT did not differ significantly in patients with sLSS between baseline and follow-up but differed significantly compared to healthy persons.

**Discussion:** The attractor for acceleration data varied largely among healthy subjects, and hence a reference attractor could not be generated. Moreover, the change in the attractor and its variability during the 6MWT differed between patients and elderly healthy persons but not between repeated assessments. Hence, the attractor based on low-pass filtered signals as used in this study may reflect pathology specific differences in gait characteristics but does not appear to be sufficiently sensitive to serve as outcome parameter of decompression surgery in patients with sLSS.

## Introduction

Human function is determined by the status of the neuromusculoskeletal system. Specifically, the interrelationship between structural aspects of the musculoskeletal and neuromuscular systems determine performance characteristics that are critical for facilitating normal movement conditions of the entire physiological range (Komi, 1984). However, many orthopedic diseases or conditions are associated with an abnormal, asymmetric, or variable gait pattern (Pirker and Katzenschlager, 2017). For instance, lumbar spinal stenosis (LSS)—a degenerative narrowing of the lumbar spinal canal—can influence mobility because of neuromuscular impairment. LSS associated radiating leg pain or pain in the lower back and/or the buttocks (Kreiner et al., 2013) frequently leads to a compromised ability to walk (Tong et al., 2007) resulting in abnormal or variable gait patterns. Symptoms in conditions affecting the neuromusculoskeletal system can be present at all times or intermittently, appear suddenly or have a creeping appearance. Hence, studying the effects of neuromuscular impairments such as those caused by LSS on ambulatory function during prolonged walking or specific functional tests such as the 6-min walk test (6MWT) provides important insights into normal and pathological neuromuscular function and performance. Yet, detailed knowledge on normal function, gait patterns, and their variability in a healthy population is a prerequisite for elucidating pathological function and gait patterns.

Patients with symptomatic LSS (sLSS) adopt strategies to avoid pain when performing daily activities such as walking that may manifest as changes in kinematic and kinetic gait parameters, and sLSS is often treated surgically by decompression surgery to relieve pain and improve mobility (Adachi et al., 2003). Patients with sLSS walk slower and with greater trunk sway, and their gait is less symmetric and generally more variable than in healthy persons (Suda et al., 2002). Moreover, cadence, stride length, gait speed, and symmetry increased and gait variability decreased in patients with sLSS treated with decompression surgery (Toosizadeh et al., 2015). However, to date it is unknown if these altered gait patterns are stable or if they change during continued walking, for instance, due to the onset of pain. The 6MWT is a standardized test first used to identify the submaximal level of functional capacity in patients with cardio-pulmonary diseases (Enright, 2003). Today, the 6MWT is commonly used for evaluating surgeries with pre- and postoperative measurements but can also capture the progress of therapeutic intervention, and has been used to assess changes in gait pattern and variability (ATS Committee on Proficiency Standards for Clinical Pulmonary Function Laboratories, 2002). Instrumented gait analysis may aid in the diagnosis by objectively revealing specific gait changes during a 6MWT associated with sLSS and monitoring rehabilitation processes after surgery.

Traditional instrumented gait analysis capturing kinematic and kinetic parameters is costly and time consuming. In recent years, gait analysis based on inertial sensors or measurement units have received increasing attention. The effective and convenient handling of inertial sensors compared to traditional multi-camera three-dimensional gait analysis may simplify and increase the efficiency of evaluating and interpreting gait data and hence is attractive for future clinical use (Tao et al., 2012). While inertial sensor data allow calculating kinematic parameters, alternative analyses based on the measured acceleration data have also been investigated. For instance, acceleration data collected by inertial sensors is considered a valid parameter for quantifying human movement (Godfrey et al., 2008). Specifically, acceleration data can be evaluated using attractor analysis, and changes in acceleration pattern and variability between conditions can be calculated and compared on an individual or group level (Vieten et al., 2013). Recently, attractor analysis has been used as an index to describe movement variability and motor fatigue in patients with multiple sclerosis (Sehle et al., 2014).

While attractor analysis of acceleration gait data may be a valuable tool for clinical applications, to date the variability in the attractor among healthy persons is unknown and data on orthopedic populations are lacking. Based on the literature, the following research questions arise:

- Is the attractor for acceleration gait data similar among healthy persons and can a reference attractor be defined?
- Does the attractor for acceleration gait data change during the 6MWT in patients with sLSS?
- Are the exercise-induced changes in the attractor in patients with sLSS greater than in healthy persons?
- Are the exercise-induced changes in the attractor affected by surgical treatment?

Answering these questions will lay the foundation for the potential use of attractor analysis of acceleration gait data as clinical tool in the assessment of diseases and conditions affecting gait and for gaining further insight into their pathomechanisms.

## Materials and methods

### Participants

Twenty-four older healthy participants [15 female; mean ± 1 standard deviation, age: 59.9 ± 10.5 years; body mass index (BMI): 24.0 ± 3.5 kg/m^2^; Table 1] were recruited from local health and sports clubs. Exclusion criteria for healthy participants were: previous surgeries or joint replacements that could influence the gait pattern; the use of walking aids; and neurological or mental disorders. Nineteen patients (8 female; age: 73.8 ± 5.3 years; BMI: 27.1 ± 4.1 kg/m^2^; Table 1) diagnosed with sLSS and scheduled for decompression surgery were included in this study. All patients were recruited between May and August 2016 before their scheduled decompression surgery. Exclusion criteria were: BMI above 35 kg/m^2^; the use of walking aids; the inability to walk for 6 min; and neurological or mental diseases. This study was approved by the regional ethics committee and performed according to the Declaration of Helsinki. All participants were informed about the study protocol and provided written consent.

**Table 1:** Mean (1 standard deviation) demographic information of participants.

**Parameter**	**Healthy**	**Patients**	***P*-value**
Sex (male/female)	9/15	11/8	
Age (years)	59.9 (10.5)	73.8 (5.3)	**<0.001**
Height (cm)	168.5 (9.7)	167.8 (9.2)	0.797
Weight (kg)	68.5 (14.8)	75.8 (9.3)	0.069
BMI (kg/m^2^)	24.0 (3.5)	27.1 (4.1)	**0.010**
**ODI (%)**		
Baseline		27.9 (16.9)	
10-week follow-up		8.5 (13.0)	
12-month follow-up		11.4 (13.5)	
**6MWD**		
Baseline	410.7 (64.3)	361.4 (100.9)	
10-week follow-up		397.5 (90.5)	
12-month follow-up		400.4 (87.4)	
D (left foot, baseline) within-subject	0.99 (0.66)^a^	1.38 (0.96)^a^	
D (left foot, baseline) between-subject	3.16	3.62	

*ODI, Oswestry Disability Index; 6MWD, 6-min walk distance; D, standard deviation of the cycles toward the attractor; ^a^standard deviation of all D-values of all subjects or patients. Bold values, statistically significant difference between groups (P < 0.05)*.

### Experimental methods

All participants completed a gait analysis with an inertial sensor gait analysis system while walking up and down a 30-m hallway for 6 min (6-min walk test, 6MWT). Healthy participants completed one gait analysis. Patients completed gait analysis on the day before decompression surgery and 10 weeks and 12 months after surgery to assess the influence of surgery on gait function. At each assessment, patients also completed the Oswestry Disability Index (ODI) Questionnaire to record their pain and perceived functional disability.

### Gait analysis during the 6MWT

For all gait analysis sessions, participants were instructed to walk up and down the same 30-m hallway for 6 min. Acceleration data were collected by the RehaGait® system (Hasomed GmbH, Magdeburg, Germany) comprising seven inertial measurement units (each comprising a triaxial accelerometer (±16 g); a triaxial gyroscope (±2000°/s); and a triaxial magnetometer (±1.3 Gs); sampling rate 400 Hz) and software provided by the manufacturer. The inertial sensors were placed on the pelvis and bilaterally on the feet, the shank, and the thigh. In our study, gait data of the first and last 30 m of the 6MWT were examined regarding changes in gait patterns and gait variability and the influence of walking exercise on gait function. After test completion, raw acceleration data were exported in csv format from the manufacturer's software. The distance walked during the 6MWT (6-min walk distance 6MWD) was recorded as a measure of gait performance.

### Oswestry disability index questionnaire (ODI)

The ODI is a self-administered valid and reliable questionnaire used to evaluate and plan further therapy and treatment options in patients with lower back pain. The ODI reflects important aspects of functional pain-related disability in activities of daily life captured by 10 items (pain intensity, personal care, lifting, walking, sitting, standing, sleeping, sexual life, social life, traveling). The sum of scores is presented in percent (0–20%—minimal disability, to 81–100%—patient bed-bound or claiming to be extremely limited by their symptoms). Changes in the ODI score can be used to monitor the patient's progression and is commonly used by physical therapists for therapy planning and patient outcome (Vianin, 2008).

### Computational methods

Attractor analysis was performed according to Vieten et al. (2013) on the raw acceleration data captured by the foot sensors of the RehaGait® system. The raw acceleration vectors were low-pass filtered at 4.5 Hz using a 4th order Butterworth filter (Woltring, 1990). The built-in software of the RehaGait^®;^ provided gait events that were used to cut the three-dimensional acceleration vectors into single strides at heel-strike. Consecutive strides were depicted as limit-cycles, where each cycle represented one stride. The attractor itself represents the mean cycle of all strides and is calculated as:

$$\begin{array}{l} {\bar{A}}_{a,C}\left(\tau_{j}\right)=\;\frac{1}{n}\sum_{i\;=\;1}^{n}{\bar{a}}_{a,C}\left(i\cdot \tau_{j}\right)+\frac{1}{n}\sum_{i\;=\;1}^{n}{\bar{b}}_{a,C}\left(t=i\cdot \tau_{j}\right)\\ \approx \;\frac{1}{n}\sum_{i\;=1}^{n}{\bar{a}}_{a,C}\left(i\cdot \tau_{j}\right). \end{array}$$

(C: beginning or end of walking test; a: right or left foot)

Three attractor parameters were defined to describe the acceleration data:

- δM describes the change in acceleration pattern between two conditions (here the first and the last 30 m of the 6MWT), and represents the difference between two attractors (greater δM corresponds to a greater change in acceleration pattern between the two conditions)
  $$\begin{array}{l} \delta M=\;\sqrt{\frac{1}{m\cdot v^{2}}\sum_{j\;=\;1}^{m}\left[{\left({\bar{A}}_{r,B}\left(\tau_{j}\right)-{\bar{A}}_{r,E}\left(\tau_{j}\right)\right)}^{2}+\;{\left({\bar{A}}_{l,B}\left(\tau_{j}\right)-\;{\bar{A}}_{l,E}\left(\tau_{j}\right)\right)}^{2}\right]} \end{array}$$

(m: number of values within the attractor; v: average speed; B: beginning; E: end of walking test; r: right foot; l: left foot)

- δD describes the change in variability around the attractor and represents the change in acceleration variability between conditions (greater δD corresponds to a greater change in variability)
  $$\begin{array}{l} \delta D=\;\sqrt{\frac{1}{m}\sum_{j\;=\;1}^{m}\left[{\left({\bar{D}}_{r,B}\left(\tau_{j}\right)-{\bar{D}}_{r,E}\left(\tau_{j}\right)\right)}^{2}{+\;\left({\bar{D}}_{l,B}\left(\tau_{j}\right)-{\bar{D}}_{l,E}\left(\tau_{j}\right)\right)}^{2}\right]} \end{array}$$
- δF is considered the attractor-based index and is the product of δM and δD.

A reference attractor was generated by calculating the mean of all attractor vectors of the control group. The standard deviation between the attractor vectors and the reference attractor was calculated to assess the between-subject variability.

### Statistical analysis

All statistical analysis and calculations were performed in SPSS Version 21 (IBM Corporation, Armonk, NY). The data was tested for normality using the Shapiro-Wilk-Test. The Mann-Whitney-U-Test was used to detect differences in attractor parameters and 6MWDs between patients and healthy subjects. Repeated measures analysis of variance (ANOVA) was performed to detect differences in attractor parameters, ODI score and 6MWD between assessments (baseline, 10-week and 12-month follow-up) within patients. Bonferroni *post-hoc* tests were applied to detect specific differences between the time points. The level of significance was set to .05 for all tests.

## Results

**Figure 1:**
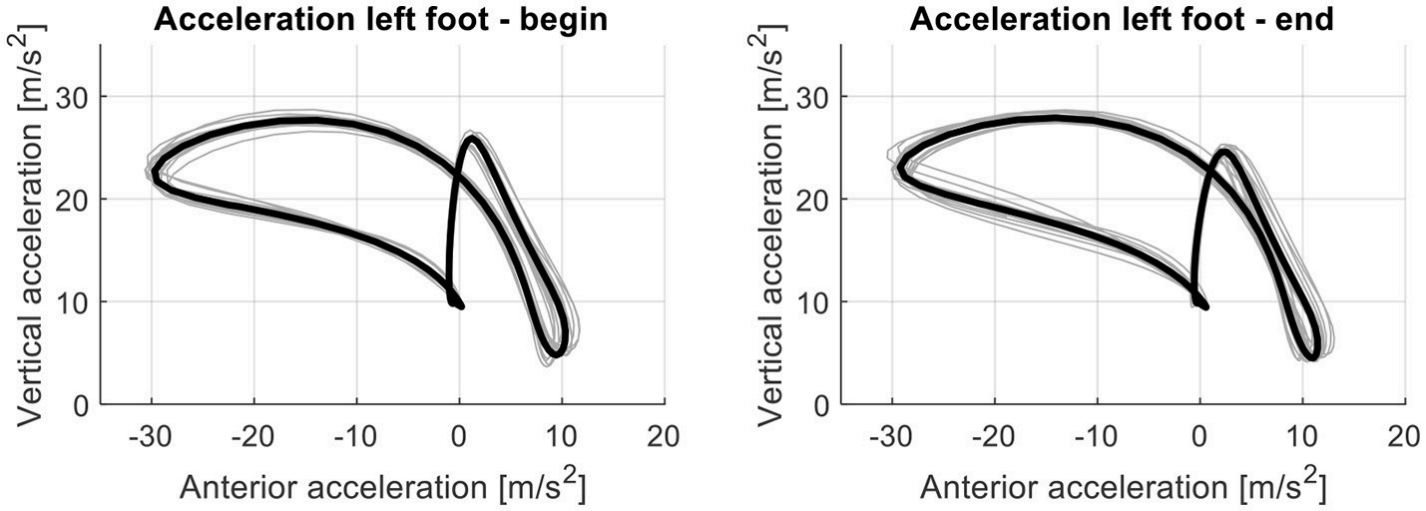
The attractor (black line; mean of acceleration loops of all steps taken during 30 m) and acceleration loops of all steps taken at the beginning (first 30 m; left graph) and end (last 30 m; right graph) of the 6MWT (gray lines) for the left foot exemplary in one healthy subject.

**Figure 2:**
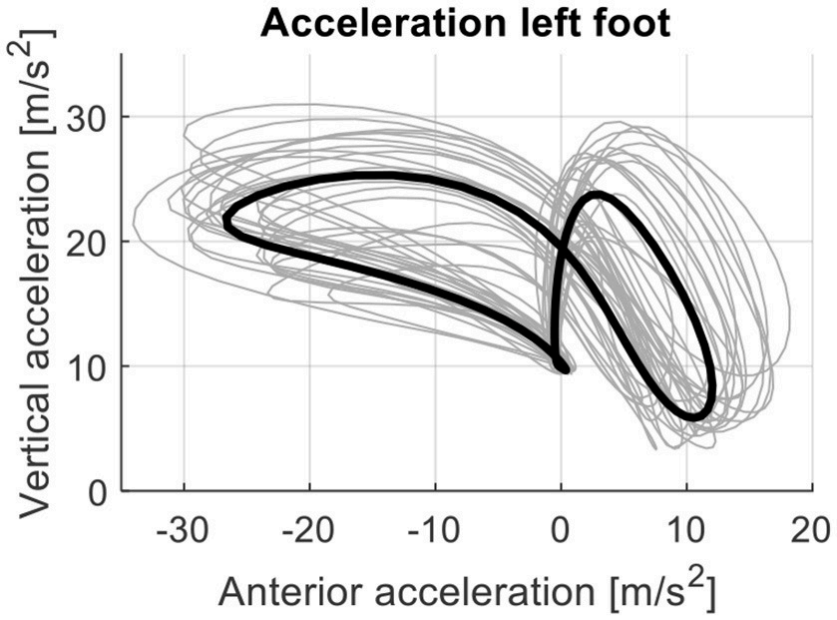
The reference attractor (black line; mean of individual attractors) and individual attractors of all healthy persons (*N* = 24; gray lines) for the left foot. Individual attractors were based on acceleration data of the foot during the first minute of the 6MWT. Differences in patterns between subjects remained after correcting for walking speed.

During the 6MWT healthy subjects walked on average a distance of 410.7 ± 64.3 m. The values of the attractor parameters and therefore the changes in acceleration pattern and variability during the 6MWT were small. The acceleration pattern and the variability around the attractor within subjects were similar for the first and last 30 m of the 6MWT (Figure 1). The attractor patterns between subjects differed greatly. The reference attractor of all healthy subjects and their individual attractors are shown in Figure 2. The between-subject variability around the reference attractor was about three times higher than the variability around the attractor within subjects (Table 1).

**Figure 3:**
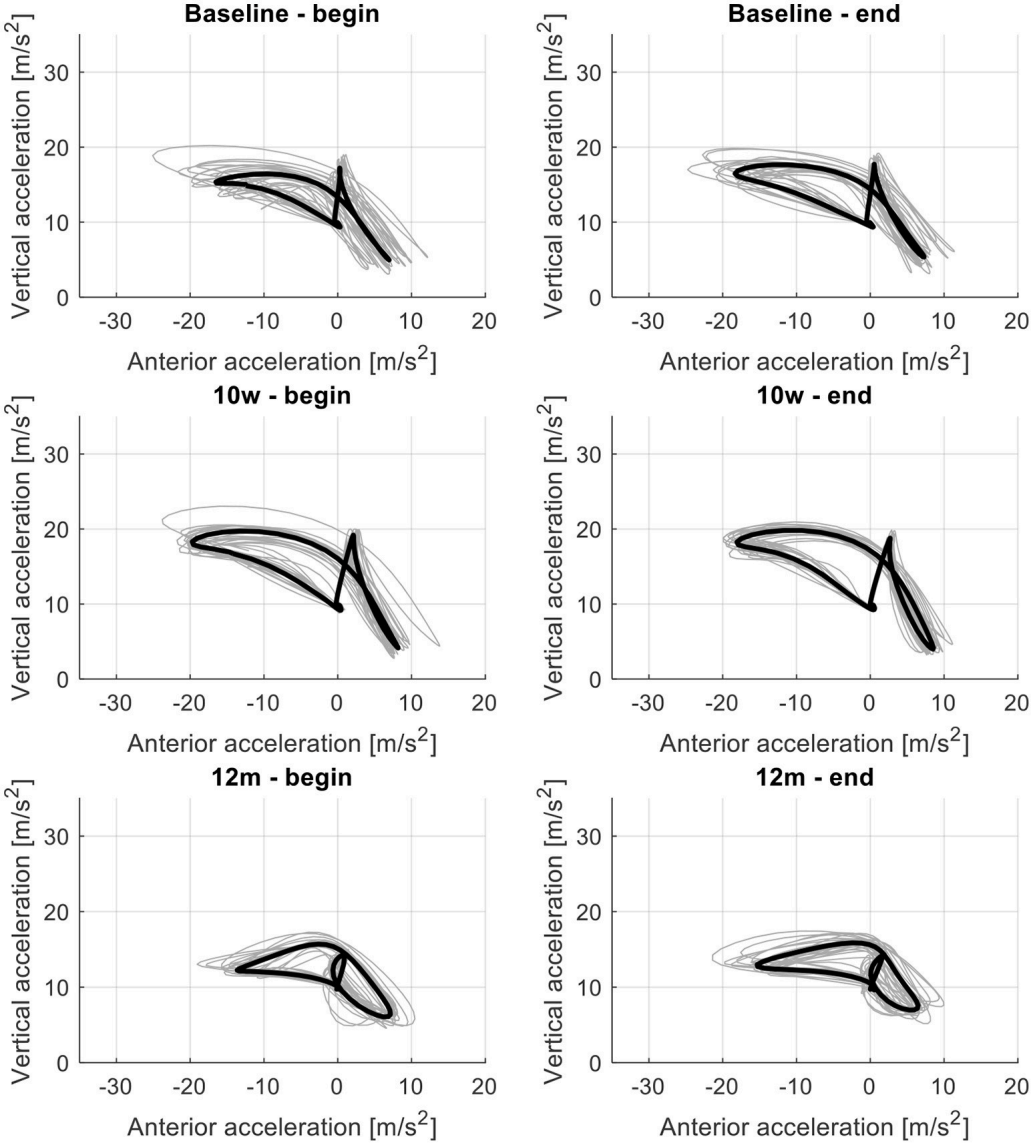
The attractor (black line; mean of acceleration loops of all steps taken during 30 m) and acceleration loops of all steps taken at the beginning (first 30 m; left graph) and end (last 30 m; right graph) of the 6-min walk test (gray lines) for the left foot for the assessments at baseline **(Top)**, 10-week follow-up **(Middle)**, and 12-month follow-up **(Bottom)** exemplary for one patient with sLSS.

The acceleration patterns and the attractor for the first and last 30 m of the 6MWT at baseline, 10-week follow-up and 12-month follow-up for one patient are shown in Figure 3. Overall, the patients showed greater changes in their gait pattern during the 6MWT than healthy persons reflected by higher δM values at all assessments compared to healthy persons (Table 2). This difference was statistically significant at the 12 month-follow-up (*P* = 0.008).

**Table 2:** Mean values and statistical results of the attractor analysis in healthy persons and patients with sLSS.

		**Norm (*N* = 24)**	**δM**	**δD**	**δF**	**6MWD (m)**		
			**2.33**	**0.79**	**2.10**	**410.7**		
		**Patients (*****N*** = **19)**	***P*****-value Norm vs. Patients**				***P*****-value patients**	**Post-hoc**
*δM*	Pre	2.66	0.127				0.087	0.839^a^; 0.344^b^;
	10w	2.99	0.169					1.00^c^
	12m	3.08	**0.008**					
*δD*	Pre	0.96		**0.007**			0.347	0.634^a^; 0.488^b^; 1.00^c^
	10w	1.24		**0.018**				
	12m	1.26		**0.001**				
*δF*	Pre	2.57			**0.007**		0.067	0.353^a^; 0.113^b^; 1.00^c^
	10w	3.70			**0.026**			
	12m	3.72			**<0.001**			
*6MWD (m)*	Pre	361.4				0.050	**0.017**	**0.002**^a^; **0.028**^b^; 1.00^c^
	10w	397.5				0.741		
	12m	400.4				0.501		
*ODI (%)*	Pre	27.9					**<0.001**	<0.001^a^; 0.001^b^;
	10w	8.5						0.810^c^
	12m	11.4						

*Statistically significant results are shown in bold (P < 0.05). δM, change in acceleration pattern; δD, change in acceleration variability; δF, attractor based index describing change in acceleration pattern and quality; pre, preoperative; 10w, 10-week follow-up; 12m, 12-month follow-up; 6MWD, 6-minute walk distance; ODI, Oswestry Disability Index score*;

a *Difference between 10-week follow-up and baseline*;

b *Difference between 12-month follow-up and baseline*;

c *Difference between 12-month follow-up and 10-week follow-up*.

The change in acceleration variability (δD) between the first and last 30 m of the 6MWT differed between patients and healthy persons (Table 2). δD values in patients were higher than those in healthy persons (*P* < 0.05 for all). The attractor-based index δF and combined value for gait quality and style (Vieten et al., 2013) was significantly greater in patients than in healthy persons at all assessments (*P* < 0.05 for all). Within the patient group, the attractor parameters did not differ significantly between assessments. Nonetheless, at the 10-week follow-up all three parameters tended to be higher than before surgery. The changes in acceleration pattern from the first to the last 30 m of the 6MWT and change in acceleration variability tended to be greater after surgery than at baseline.

The difference in mean 6MWD between patients and healthy persons decreased from 49.3 m pre-operatively to 13.2 m at 10 weeks to 10.3 m at 12 months although these differences were not significant (Tables 1, 2). These differences in gait performance, parameters, and variability persisted after correcting for age and BMI.

## Discussion

The objectives of this study were to determine if the attractor for acceleration gait data is similar among healthy persons defining a reference attractor; if exercise-induced changes in the attractor in patients with sLSS are greater than in healthy persons; and if the exercise-induced changes in the attractor are affected by surgical treatment. Our results showed that the attractor for acceleration gait data varies largely among healthy subjects, and hence a reference attractor cannot be defined. Moreover, the change in the attractor and its variability during the 6MWT differed between patients and elderly healthy persons but not between repeated assessments. Hence, the attractor may reflect pathology specific differences in gait characteristics but does not appear to be sufficiently sensitive to serve as outcome parameter of decompression surgery in patients with sLSS.

Interestingly, we observed a large variability in the attractor for acceleration gait data among healthy subjects that was much greater than the intra-individual variability in acceleration patterns. Inter-subject variability in attractor patterns remained unaffected by normalization to walking speed. This result is particularly interesting because walking faster requires higher foot acceleration after the stationary period during ground contact. However, our results showed that foot acceleration patters are highly individual and may in fact be considered a person's unique “foot acceleration print.” Moreover, acceleration patterns and their variability were similar at the beginning and end of the 6MWT indicated by small attractor parameters. These results suggest that acceleration patterns are stable throughout a non-fatiguing exercise in healthy persons. Based on these results we postulate that

- there is no reference attractor on foot acceleration data characterizing normal gait; and
- individual attractor patterns on foot acceleration may be a unique characteristic of a person's gait.

This result is in agreement with a recent study by Broscheid et al. (2018) who have shown that the fundamental walking pattern described by the acceleration attractor does not change with rehabilitation or after a single training session. The fact that correcting attractor patterns for walking speed did not reduce the variability in patterns between persons leads to the speculation that this pattern may be invariant to changes in walking speed associated with aging (Frimenko et al., 2015). This result may open up new opportunities in identifying groups of individuals who respond differently to intervention or being at higher risk for incurring an injury or disease. Factors that could contribute to the large variability include parameters that cannot be influenced such as sex or body height and parameters that can be modified such as body mass or parameters of neuromuscular performance (e.g., muscle strength or muscle coordination). For instance, Akalan et al. (2016) have shown that iliopsoas muscle group weakness resulted in related hip joint velocity reduction and stiff-knee gait during walking in healthy persons. However, the paradigm of a unique “foot acceleration print” similar to the previously proposed gait print (Broscheid et al., 2018) warrants further investigation.

At all assessments, the change in acceleration variability and in the attractor index during the 6MWT was greater in patients with sLSS than in healthy subjects. This result indicates that a 6MWT is sufficient to elicit functional changes in patients with sLSS. Changes in gait stability during the 6MWT have been previously reported in patients after stroke (Iosa et al., 2012). In another study on the same subjects and patients, we did not observe changes in spatiotemporal gait parameters and gait asymmetry during the 6MWT. Hence, it appears that attractor analysis on foot acceleration data is more sensitive for detecting changes in gait patterns during a relatively short functional gait test than traditional gait parameters. The attractor analysis implemented in our study was based on previous research of Sehle et al. (2014) who defined a fatigue index for patients with multiple sclerosis. In their study, patients were asked to walk on a treadmill until complete exhaustion, which occurred in less than 30 min in all patients, while healthy subjects were asked to walk for 30 min. The attractor index in our patients was below the fatigue index cut-off for motor fatigue (δ*F* = 4) reported by Sehle et al. (2014). Because we limited the walking exercise to 6 min based on the widely accepted use of the 6MWT in clinical cohorts (Gao et al., 2014; Dunn et al., 2015; O'Brien et al., 2016; Keilani et al., 2017; Withers et al., 2017), it is remarkable that we still observed greater changes in attractor variability in patients than in healthy persons. It is unknown if patients in our study would have experienced even greater changes in attractor variability if they would have continued to walk until exhaustion or inability to continue due to sLSS symptoms. However, smaller attractor index values in our population are coherent with clinical observations of poorer gait function after exhaustion in patients with multiple sclerosis and motor fatigue than in patients with sLSS.

On average, patients with sLSS benefitted from decompression surgery: the ODI score decreased significantly from baseline to 10 weeks after decompression surgery and remained unchanged until 12 months after surgery. Higher ODI scores indicate less mobility and more pain. Thus, on average the decompression surgery reduced pain and symptoms. Similar results were found by McGirt et al. (2015), where the ODI improved significantly 12 months post-operatively. Moreover, the 6MWD improved significantly after decompression surgery in patients with sLSS from values below those of healthy persons to values similar to those in healthy persons. These results are in agreement with reports of improved functional capacity after decompression surgery (Smuck et al., 2018). This result demonstrates the importance of decompression surgery for regaining quality of life (Zarghooni et al., 2017).

In contrast to improvements in gait performance, exercise-induced changes in acceleration patterns and variability did not differ between assessments. We had expected that the exercise-induced changes in acceleration pattern and variability would decrease after decompression surgery. There are several possible explanations for this result. Because of the long duration of symptoms (at least 6 months) patients may have adopted a gait pattern preoperatively to compensate for pain characterized by greater variability that was unaffected by decompression surgery. Moreover, the long duration of symptoms and the claudication was likely associated with compromised muscle strength, impairment in trunk extensor muscle endurance, leg strength, leg strength asymmetry, or passive knee and ankle range of motion (Schmidt et al., 2017). Schmidt et al. also showed that impairment in trunk extensor muscle endurance, leg strength, leg ROM, and asymmetry of strength and ROM are associated with performance-based mobility (Schmidt et al., 2017).

The lack of statistically significant differences in exercise-induced changes in acceleration patterns and variability over time may be attributed to the heterogeneity of our patient population. We enrolled patients in our study independent of the type of sLSS. Hence, future studies may not only further explore the association between neuromuscular attributes and exercise-induced changes in acceleration patterns and variability but also relate this association to location of claudication based on medical imaging. Moreover, although not reported here, comorbidities present in some patients may have influenced their gait patterns independent of the limitations caused by the LSS. Further, the age of the patients may have played a role because geriatric patients experience skeletal and muscular changes (Marzetti and Leeuwenburgh, 2006) that can influence the patients' gait and neuromuscular attributes and therefore influence attractor patterns. However, including age in our statistical models did not affect the results. Nonetheless, this possibility could be further explored to investigate the pathomechanism of sLSS. Moreover, in contrast to treadmill walking assessed by Sehle et al. (2014), we assessed foot accelerations during overground walking. For instance, Bizovska et al. (2018) have shown that stride time variability and short-term Lyapunov exponents of acceleration data in all directions are greater for overground than for treadmill walking. However, it remains unknown if the change in acceleration variability would also depend on the walking condition.

In summary, we explored the use of attractor analysis on low-pass filtered acceleration data to assess changes in acceleration patterns and variability during a defined walking exercise. We presented answers to our research questions:

- Is the attractor for acceleration gait data similar among healthy persons and can a reference attractor be defined? We observed a large variability in attractors of acceleration patterns among healthy subjects precluding the definition of a reference attractor.
- Does the attractor for acceleration gait data change during the 6MWT in patients with sLSS? The change in the attractor and its variability during the 6MWT did not differ between repeated assessments.
- Are the exercise-induced changes in the attractor in patients with sLSS greater than in healthy persons? In general, patients had greater exercise-induced changes in acceleration patterns than healthy persons.
- Are the exercise-induced changes in the attractor affected by surgical treatment? Exercise-induced changes in acceleration patterns were not affected by decompression surgery.

Hence, overall patients had a less stable gait than healthy persons pre- and postoperatively. Multiple factors may play an important role for the difference between patients and the control group, such as the age of participants or other coexisting diseases, or secondary changes of sLSS including compromised neuromuscular performance. Readjusting gait patterns after surgery may require more than 12 months as indicated by a trend toward increasing changes in acceleration patterns post-operatively.

## Ethics statement

This study was carried out in accordance with the recommendations of Swissethics and Ethics Committee Northwest/Central (EKNZ). The protocol was approved by the Swissethics and Ethics Committee Northwest/Central (EKNZ). All subjects gave written informed consent in accordance with the Declaration of Helsinki.

## Author contributions

SB, AM, and CNü conceived the study. SB, AL, and SL collected the data. SB and CNü processed the data. SB, AM, and CNü analyzed the data. All authors were involved in data interpretation. SB wrote the manuscript. All authors critically revised the manuscript and approved of the final version.

### Conflict of interest statement

The authors declare that the research was conducted in the absence of any commercial or financial relationships that could be construed as a potential conflict of interest. The handling Editor declared a past co-authorship with the authors.
